# Mechanisms of Cell Damage in Neurological Diseases and Putative Neuroprotective Strategies

**DOI:** 10.1155/2018/9784319

**Published:** 2018-06-25

**Authors:** Perla D. Maldonado, María Elena Chánez-Cárdenas, Arsenio Fernández-López

**Affiliations:** ^1^National Institute of Neurology and Neurosurgery, Mexico City, Mexico; ^2^Area de Biologia Celular, Instituto de Biomedicina, Universidad de Leon, León, Spain

The pathophysiology of neurological disorders often involves deleterious changes in cellular homeostasis. For example, several diseases including Alzheimer's, Parkinson's, Huntington's, acquired immunodeficiency syndrome- (AIDS-) dementia complex, or hepatic encephalopathy can compromise cellular functions through excitotoxicity, cytoplasmic Ca^2+^ overload, overproduction of reactive oxygen and nitrogen species, and overall energy failure and inflammation. Alterations in cellular energy levels, redox balance, and Ca^2+^ levels are also known to reduce the protein folding capacity of the endoplasmic reticulum. This in turn leads to the accumulation and aggregation of unfolded proteins, a condition known as endoplasmic reticulum stress. All these cellular events result in different types of regulated cell death in the cerebral tissue. The pathophysiological mechanisms that lead to neuronal injury in neurological disorders are complex and multifactorial. Consequently, the development of preclinical testing strategies to study these mechanisms is relevant in order to find new therapeutic targets for the treatment of these debilitating diseases.

The study of different neuroprotective mechanisms in different cerebral damage models, both *in vivo* and *in vitro*, is a very active area of research. This special issue includes state-of-the-art models of cerebral ischemia, in vitro primary cerebellar granule neurons, traumatic brain injury and spinal cord ischemia-reperfusion to explore neuroprotective strategies and compounds. The original research and review articles in this issue describe pathophysiological mechanisms involved in the cellular response to damage such as the role of autophagy, apoptosis, NADPH oxidase inhibition, oxidative stress, anti-inflammatory activity, and mitochondrial metabolism. The role of astrocytes and microglia in neuroprotection is also discussed.

Autophagy is involved in the breakdown of damaged organelles and misfolded proteins via a stress-induced catabolic pathway to maintain the cellular homeostasis. However, it does not always result in cell survival. In spinal cord ischemia-reperfusion (SCIR), autophagy is upregulated; but whether it plays a protective or a neurodegenerative role is controversial. In this special issue, L. Xie et al. report that oxidative stress is the main trigger of autophagic cell death during SCIR injury. They also show that hydrogen sulfide treatment exerts a neuroprotective effect by reducing oxidative stress. These data suggest a potential application of hydrogen sulfide in the SCIR damage. On the other hand, C. Cui et al. using the active metabolite of vitamin D, calcitriol, in a traumatic brain injury model, also report neuroprotective effects. They describe that the protection of calcitriol occurs through the downregulation of NADPH oxidase, activation of vitamin D receptor expression, and the suppression of apoptosis in the CA1 region of the hippocampus.

The understanding of the pathogenesis of stroke has shown dramatic advances; however, clinical trials have resulted in negative results after evaluating numerous promising compounds. Thus, the active search for new compounds continues, and new agents are assayed. In this issue, Z. Wang et al. designed and synthesized novel twin compounds containing tetramethylpyrazine and carnitine substructures. They found that LR134 and LR143 compounds induced important neuroprotection by reducing cerebral infarct and edema, while improving the neurological function and the blood-brain barrier integrity after cerebral ischemia/reperfusion injury. The protective effect observed was associated with a reduced inflammatory response and a decrease in NADPH oxidase-mediated oxidative stress. They also report an improvement of energy supply. Their data suggest that these chemical structures may represent an innovative therapeutic strategy for patients with stroke.

Cerebral ischemia triggers a cell-specific cascade of events, leading to neuronal death. Neurons, astrocytes, microglia/macrophages, neutrophils, endothelial cells, and platelets exhibit different functional roles after brain ischemia/reperfusion injury, making it difficult to know the role of each of these cell types. In this context, S. Y. Cheon et al. present the role of apoptosis signal-regulating kinase 1 (ASK1) in different cell types suggested by preclinical studies and the potential use of ASK suppression (pharmacologic or genetic), as a promising therapeutic option for ischemic stroke recovery. In addition, R. Thakkar et al. examine the ability of 17 beta-estradiol (E2) to regulate the activation of microglia phenotype in the hippocampus using a global cerebral ischemia model (GCI). They show that after GCI, E2 exerts a neuroprotective effect, promoting the anti-inflammatory microglia phenotype in the hippocampus.

A. N. Winter et al. used primary cultures of cerebellar granular neurons subjected to hydrogen peroxide-induced oxidative stress to test 4-hydroxybenzoic acid (HBA) and protocatechuic acid (PCA). They report that PCA plays a neuroprotective role during inflammation conditions, while HBA protects under conditions of excitotoxicity. Additionally, PCA promotes anti-inflammatory activity in microglial cells stimulated with lipopolysaccharide reducing nitric oxide production.

Finally, M. A. Bylicky et al. propose that astrocytes play critical functions for the maintenance and protection of neurons under conditions of acute or chronic injury. These functions rely on specialized responses under stress conditions. Therefore, the understanding of the mechanisms used by the astrocytes to protect the brain will allow the development of novel therapeutic pathways to protect neurons in conditions of acute injury.

The papers published in this special issue show the complexity of the mechanisms involved in the pathophysiology of neurological diseases, while highlighting the still current limited treatment options as well as the urgent need for the development of preclinical studies to find effective therapies against these pathologies.

*Perla D. Maldonado* *María Elena Chánez-Cárdenas* *Arsenio Fernández-López*

